# Optimizing Patient Outcomes in Spinal Surgery: An Investigation Into Anesthesiologists’ Case Volume

**DOI:** 10.7759/cureus.49559

**Published:** 2023-11-28

**Authors:** Parimal Rana, Jane C Brennan, Andrea H Johnson, Justin J Turcotte, Chad Patton

**Affiliations:** 1 Surgical Research, Luminis Health Anne Arundel Medical Center, Annapolis, USA; 2 Orthopedics, Luminis Health Anne Arundel Medical Center, Annapolis, USA; 3 Orthopedic Surgery, Luminis Health Anne Arundel Medical Center, Annapolis, USA

**Keywords:** lumbar discectomy, lumbar-fusion, anterior cervical discectomy and fusion (acdf), surgical case volume, spine surgery anesthesia

## Abstract

Introduction

Nearly one million patients in the United States undergo spine surgical procedures annually to seek relief from chronic back and neck pain. A multidisciplinary approach is key to ensuring the efficiency and safety of the surgical process, with the anesthesia team, nursing, surgeon, and healthcare facilities all playing a role. The purpose of this study is to capture potential associations between the anesthesiologists' case volume and patient postoperative outcomes in the early recovery period.

Methods

A retrospective review of anterior cervical discectomy and fusion (ACDF), lumbar decompression (LD), and lumbar fusion (LF) patients from July 2019 to June 2023 was performed. Anesthesiologists were categorized into low, medium, and high volumes of spine surgical cases. Univariate analysis was performed on patient demographics, intraoperative measures, post-anesthesia care unit (PACU) measures, and postoperative measures by anesthesiologist volume.

Results

This study included 545 ACDF, 815 LD, and 1,144 LF patients. There were no differences between groups in ACDF patients by anesthesiologist volume. When examining patients undergoing LD, there was a difference in patients with an American Society of Anesthesiologists (ASA) physical status classification of three or greater (low volume: 41.7% vs. medium volume: 53.7% vs. high volume: 45.0%; p=0.029). When examining patients undergoing LF, there were differences in patients with low temperatures in PACU (low volume: 2.8% vs. medium volume: 7.3% vs. high volume: 4.2%; p=0.044) and the percentage of patients with a 90-day emergency department return (low volume: 7.7% vs. medium volume: 11.9% vs. high volume: 7.0%; p=0.024).

Conclusion

While this study found a minimal impact of anesthesiologist volume on postoperative outcomes, recent literature has emphasized the critical role of teamwork and specialized surgical teams to enhance efficiency and patient care. Further studies are warranted to identify other variables in anesthesia, nursing, and surgical team workflow that may impact postoperative outcomes in spinal surgeries.

## Introduction

An estimated 10-15% of the population suffers from chronic back pain, with approximately 900,000 Americans utilizing spine surgery annually to seek relief [1]. Spine surgery often demands a multidisciplinary approach for optimal patient outcomes. Central to this collaborative effort are the anesthesia team, nursing staff, surgeon, and healthcare facilities, each playing a vital role in ensuring the efficiency and safety of the surgical process. While individual expertise at every level is unquestionably imperative, it is the seamless coordination, proficiency, and interaction among these components that ultimately determine the success of the procedure.

In recent years, studies have delved into the impact of surgical volume and dedicated teams on the outcomes of spinal surgeries. Notably, the investigation conducted by Martin et al. and Dony et al. assessed the effects of dedicated anesthesia care teams, unveiling substantial enhancements in the quality of care when a dedicated team system was implemented [2,3]. Having a standardized anesthesia protocol can help produce successful spine and other orthopedic surgery outcomes by decreasing variability between providers; however, the literature on the impact of the volume of cases of the anesthesiologist on outcomes is limited. Thus, our investigation aims to capture potential associations with anesthesiologists' case volume and patient postoperative outcomes in the post-anesthesia care unit (PACU) and early recovery periods.

## Materials and methods

Study population

The study was performed at Luminis Health Anne Arundel Medical Center, Annapolis, Maryland, USA. The institutional review board deemed this study exempt. A retrospective review of 545 anterior cervical discectomy and fusions (ACDF), 1,144 lumbar fusions (LF), and 814 lumbar decompressions (LD) from July 1, 2019, to June 30, 2023, was performed. Patient demographics, comorbidities, procedure performed, PACU measures, length of stay, and postoperative outcomes were collected.

Primary outcomes

The primary outcomes of interest were the following PACU measures and postoperative outcomes: Pasero Opioid-induced Sedation Scale (POSS) 4 in PACU, Numeric Rating Scale (NRS) pain score greater than or equal to seven in PACU, low temperature (<36 °C) in PACU, reintubation in PACU, nausea in PACU, urinary retention requiring a Foley catheter in PACU, minutes in recovery, length of stay (hours and days), non-home discharge, 90-day emergency department (ED) return, and 90-day readmission.

Statistical analysis

Anesthesiologists were categorized by the volume of spinal surgeries into low, medium, and high classes. Anesthesia volume classification was determined by tertiles; the first tertile was one to seven surgeries, the second was eight to 11 surgeries, and the third was 12 or more surgeries. From 2019 to 2023, anesthesiologists with up to seven spinal surgeries were low volume, eight to 11 surgeries were medium volume, and 12 or more surgeries were high volume. Volume for each individual anesthesiologist was defined as the average number of spine cases performed per year during the study time period. Univariate analysis, including chi-square tests and Kruskal-Wallis tests, was used to determine differences in patient demographics, comorbidities, procedure performed, PACU measures, length of stay, and postoperative outcomes by anesthesiologist volume for each procedure. All statistical analyses were performed using R Studio (Version 4.2.2 © 2009-2023 RStudio, PBC, Boston, United States). Statistical significance was assessed at p<0.05.

Source of funding

This study did not receive any funding.

## Results

Of the 545 ACDFs, the average patient age was 58 years old, the average body mass index (BMI) was 30 kg/m2, 293 (54%) patients were female, 254 (47%) patients had an American Society of Anesthesiologists (ASA) physical status classification of three or greater, 100 (18%) were non-white, and seven (1%) were Hispanic. The average number of levels operated on was 2.5, and the average time in the operating room (OR) was 168 minutes. In the PACU, 30 (6%) had a POSS of four, 203 (37%) had a NRS pain score of seven or greater, eight (1)% had a temperature below 36 °C, 22 (4%) required reintubation, 22 (4%) had nausea, and seven (1%) had urinary retention. The average time in recovery was 168 minutes. Postoperatively, the average length of stay was 1.5 days; 18 (3%) patients were not discharged home; 40 (7%) patients returned to the ED within 90 days; and 13 (2%) were readmitted within 90 days postoperatively. When comparing by anesthesiologist volume, there were no significant differences in any of these measures (Table 1).

**Table 1:** Demographic, procedure, and postoperative details of anterior surgical discectomy and fusion by anesthesiologist volume All data presented as mean ± SD or n (%); statistical significance p<0.05 in bold; ASA: American Society of Anesthesiologists; POSS: Pasero Opioid-induced Sedation Scale; NRS: Numeric Rating Scale; PACU: post-anesthesia care unit; ED: emergency department

	Low Volume (n=70)	Medium Volume (n=177)	High Volume (n=298)	P-Value
Demographics				
Age, years	60.1 ± 12.9	57.0 ± 12.1	58.4 ± 11.3	0.176
Body mass index, kg/m^2^	29.8 ± 5.96	30.6 ± 6.43	30.6 ± 6.16	0.776
Female	39 (55.7)	94 (53.1)	160 (53.7)	0.933
Non-white race	10 (14.3)	29 (16.4)	61 (20.5)	0.309
Hispanic	0 (0)	5 (2.8)	2 (0.7)	0.083
ASA score ≥ 3	31 (44.3)	82 (46.3)	141 (47.3)	0.926
Procedure				
Number of levels	2.61 ± 1.04	2.50 ± 1.21	2.56 ± 1.00	0.405
Surgeon				0.504
1	17 (24.3)	40 (22.6)	85 (28.5)	
2	3 (4.3)	3 (1.7)	4 (1.3)	
3	9 (12.9)	38 (21.5)	56 (18.8)	
4	3 (4.3)	3 (1.7)	11 (3.7)	
5	12 (17.1)	32 (18.1)	44 (14.8)	
6	6 (8.6)	22 (12.4)	37 (12.4)	
7	20 (28.6)	39 (22.0)	61 (20.5)	
Minutes in the operating room	168.8 ± 49.8	172.5 ± 68.7	165.1 ± 53.2	0.770
Post-anesthesia care unit				
POSS 4 in PACU	4 (5.7)	7 (4.0)	19 (6.4)	0.533
NRS pain score ≥ 7 in PACU	28 (40.0)	66 (37.3)	109 (36.6)	0.868
Low temp in PACU	2 (2.9)	2 (1.1)	4 (1.3)	0.575
Reintubation	1 (11.4)	6 (3.4)	15 (5.0)	0.335
Nausea in PACU	4 (5.7)	8 (4.5)	10 (3.4)	0.615
Urinary retention	2 (2.9)	1 (0.6)	4 (1.3)	0.351
Minutes in recovery	171.7 ±61.2	160.7 ±84.4	170.8± 73.5	0.110
Postoperative outcomes				
Length of stay, hours	39.8 ± 36.2	40.4 ± 53.5	46.3± 92.6	0.427
Length of stay, days	1.36 ±1.56	1.40 ± 2.27	1.66 ±3.88	0.426
Non-home discharge	2 (2.9)	4 (2.3)	12 (4.0)	0.567
90-day ED return	8 (11.4)	12 (6.8)	20 (6.7)	0.372
90-day readmission	2 (2.9)	5 (2.8)	6 (2.0)	0.823

Of the 815 lumbar decompressions, the average patient age was 60 years old, the average BMI was 30 kg/m2, 340 (42%) patients were female, 111 (14%) were non-white, and 19 (2%) were Hispanic. The average number of levels operated on was 1.5, and the average time in the OR was 121 minutes. In the PACU, 39 (5%) had a POSS of four, 140 (17%) had a NRS pain score of seven or greater, 18 (2%) had a temperature below 36 °C, 40 (5%) required reintubation, 36 (4%) had nausea, and 28 (3%) had urinary retention. The average time in recovery was 114 minutes. Postoperatively, the average length of stay was 1.2 days; 38 (5%) of patients were not discharged home; 65 (8%) patients returned to the ED within 90 days; and 13 (2%) were readmitted within 90 days postoperatively. When comparing by anesthesiologist volume, the only significant difference was the percentage of patients with an ASA of 3 or greater (low volume: 41.7% vs. medium volume: 53.7% vs. high volume: 45.0%; p=0.029) (Table 2).

**Table 2:** Demographic, procedure, and postoperative details of lumbar decompression by anesthesiologist volume All data presented as mean ± SD or n (%); statistical significance p<0.05 in bold; ASA: American Society of Anesthesiologists; POSS: Pasero Opioid-induced Sedation Scale; NRS: Numeric Rating Scale; PACU: post-anesthesia care unit; ED: emergency department

	Low Volume (n=108)	Medium Volume (n=283)	High Volume (n=424)	P-Value
Demographics				
Age, years	59.7 ± 16.7	62.4 ± 15.1	59.3 ± 16.1	0.063
Body mass index, kg/m^2^	31.0 ± 7.1	31.1 ± 6.6	30.5 ± 6.8	0.489
Female	43 (39.8)	121 (42.8)	176 (41.5)	0.863
Non-white race	16 (14.8)	40 (14.1)	55 (13.0)	0.815
Hispanic	1 (0.9)	8 (2.8)	10 (2.4)	0.564
ASA score ≥ 3	45 (41.7)	152 (53.7)	191 (45.0)	0.029
Procedure				
Number of levels	1.32 ± 0.7	1.44 ± 0.8	1.49 ± 0.9	0.288
Surgeon				0.206
1	6 (5.6)	10 (3.5)	29 (6.8)	
2	5 (4.6)	10 (3.5)	11 (2.6)	
3	12 (11.1)	27 (9.5)	43 (10.1)	
4	4 (3.7)	6 (2.1)	20 (4.7)	
5	36 (33.3)	83 (29.3)	105 (24.8)	
6	24 (22.2)	92 (32.5)	116 (27.4)	
7	21 (19.4)	55 (19.4)	100 (23.6)	
Minutes in operating room	123.5 ± 47.0	120.6 ± 35.5	121.1 ± 40.4	0.638
Post-anesthesia care unit				
POSS 4 in PACU	4 (3.7)	16 (5.7)	19 (4.5)	0.659
NRS pain score ≥ 7 in PACU	15 (13.9)	57 (20.1)	68 (16.0)	0.228
Low temp in PACU	0 (0)	8 (2.8)	11 (2.6)	0.222
Reintubation	7 (6.5)	13 (4.6)	20 (4.7)	0.717
Nausea in PACU	5 (4.6)	13 (4.6)	18 (4.2)	0.969
Urinary retention	4 (3.7)	9 (3.2)	15 (3.5)	0.955
Minutes in recovery	106.8 ± 60.3	115.8 ± 68.3	115.3 ± 61.9	0.457
Postoperative outcomes				
Length of stay, hours	28.6 ± 41.9	34.7 ± 65.6	38.4 ± 112.0	0.793
Length of stay, days	0.94 ± 1.78	1.17 ± 2.76	1.34 ± 4.71	0.468
Non-home discharge	2 (1.9)	17 (6.0)	19 (4.5)	0.212
90-day ED return	7 (6.5)	24 (8.5)	34 (8.0)	0.807
90-day readmission	0 (0)	7 (2.5)	8 (1.9)	0.265

Of the 1,144 lumbar fusions, the average patient age was 63 years old, the average BMI was 31 kg/m2, 649 (57%) patients were female, 621 (54%) patients had an ASA of three or greater, 177 (15%) were non-white, and 29 (3%) were Hispanic. The average number of levels operated on was 2.4, and the average time in the OR was 252 minutes. In the PACU, 115 (10%) had a POSS of 4, 455 (40%) had a NRS pain score of seven or greater, 69 (6%) required reintubation, 62 (5%) had nausea, and 55 (5%) had urinary retention. The average time in recovery was 193 minutes. Postoperatively, the average length of stay was 2.8 days, 117 (10%) patients were not discharged home, and 48 (4%) were readmitted within 90 days postoperatively. When comparing anesthesiologist volume, there were significant differences in the percentage of patients with low temperatures in the PACU (low volume: 2.8% vs. medium volume: 7.3% vs. high volume: 4.2%; p=0.044) and the percentage of patients who return to the ED within 90 days postoperatively (low volume: 7.7% vs. medium volume: 11.9% vs. high volume: 7.0%; p=0.024) (Table 3).

**Table 3:** Demographic, procedure, and postoperative details of lumbar fusion by anesthesiologist volume All data presented as mean ± SD or n (%); statistical significance p<0.05 in bold; ASA: American Society of Anesthesiologists; POSS: Pasero Opioid-induced Sedation Scale; NRS: Numeric Rating Scale; PACU: post-anesthesia care unit; ED: emergency department

	Low Volume (n=142)	Medium Volume (n=386)	High Volume (n=616)	P-Value
Demographics				
Age, years	63.0 ±12.3	62.8± 12.7	63.4 ±11.9	0.920
Body mass index, kg/m^2^	31.3 ±5.7	30.6± 5.9	30.9± 5.9	0.335
Female	73 (51.4)	224 (58.0)	352 (57.1)	0.378
Non-white race	17 (12.0)	59 (15.2)	101 (16.4)	0.423
Hispanic	4 (2.8)	10 (2.6)	15 (2.4)	0.968
ASA score ≥ 3	70 (49.3)	200 (51.8)	351 (57.0)	0.146
Procedure				
Number of levels	2.68 ±1.88	2.26 ±1.49	2.36 ±1.48	0.057
Surgeon				0.367
1	25 (17.6)	81 (21.0)	129 (20.9)	
2	2 (1.4)	14 (3.6)	25 (4.1)	
3	17 (12.0)	44 (11.4)	65 (10.6)	
4	8 (5.6)	4 (1.0)	17 (2.8)	
5	31 (21.8)	93 (24.1)	144 (23.4)	
6	32 (22.5)	83 (21.5)	136 (22.1)	
7	27 (19.0)	67 (17.4)	100 (16.2)	
Minutes in operating room	261.3 ±104.9	249.5 ±104.4	250.4 ±94.9	0.185
Post-anesthesia care unit				
POSS 4 in PACU	15 (10.6)	37 (9.6)	63 (10.2)	0.926
NRS pain score ≥ 7 in PACU	57 (40.1)	154 (39.9)	244 (39.6)	0.991
Low temp in PACU	4 (2.8)	28 (7.3)	26 (4.2)	0.044
Reintubation	11 (7.7)	26 (6.7)	32 (5.2)	0.399
Nausea in PACU	9 (6.3)	22 (5.7)	31 (5.0)	0.789
Urinary retention	9 (6.3)	18 (4.7)	28 (4.5)	0.658
Minutes in recovery	187.9 ±107.8	193.3 ±108.0	194.7 ±95.4	0.485
Postoperative outcomes				
Length of stay, hours	88.8 ±124.3	74.1± 83.1	70.2± 59.0	0.396
Length of stay, days	3.39 ±5.19	2.78 ±3.50	2.60 ±2.47	0.356
Non-home discharge	17 (12.0)	43 (11.1)	57 (9.3)	0.483
90-day ED return	11 (7.7)	46 (11.9)	43 (7.0)	0.024
90-day readmission	4 (2.8)	13 (3.4)	31 (5.0)	0.301

## Discussion

Achieving efficiency and safety while decreasing negative outcomes in spine surgery relies on a complex interplay of factors involving the surgeon, anesthesia team, nursing staff, and healthcare facilities. The present study revealed that anesthesiologist volume has no significant impact on the differences in PACU measures, length of stay, or postoperative outcomes when comparing anesthesiologists with different surgical volumes. It is worthwhile to note that in lumbar fusion cases, high-volume anesthesiologists saw a lower 90-day ED return. Nevertheless, prior research has demonstrated additional elements within the complex surgical process that can impact the quality of patient outcomes.

Teamwork has emerged as a critical factor in ensuring efficient and safe patient care in recent literature, emphasizing the importance of collaborative efforts in surgical settings. Studies have shown that procedures performed by teams of anesthesiologists and anesthesia nurses are associated with reduced postoperative mortality and shorter hospital stays compared to those performed by solo anesthesiologists [3]. Effective anesthesia monitoring in spine surgery significantly impacts outcomes as well, with teams having less than 100 cases of experience exhibiting over twice the postoperative neurological complication rate compared to more experienced teams [4]. These studies highlight the benefits of a coordinated approach in the perioperative period, and the implementation of a dedicated anesthesia team model demonstrates the positive impact of specialized teams on surgical efficiency.

A comparative study of quality perceptions and preoperative efficiency across institutions in spine surgery found the role of surgical staff awareness and proficiency in preoperative tasks specific to the procedure was significant to the quality of care produced. They found larger university hospitals had inconsistent nursing and technician assignments to procedures, whereas private and smaller hospitals were able to assign the same team on a regular basis [5]. A consistent team not only reduced preoperative time spent in the OR but also minimized fluoroscopy radiation as the team had greater awareness of the procedure and required tasks.

In the context of adolescent scoliosis patients undergoing posterior spinal fusion (PSF), Martin et al. found having a dedicated spine team resulted in decreased surgical and total OR time, reduced blood loss, and lower transfusion rates [2]. Flynn et al. also found that having dedicated PSF spine teams allowed members to develop standardized protocols and techniques for patient transport, positioning, preparation, draping, imaging, and recovery. In addition to a reduction in OR time by two hours, they found a cost reduction of $6000-8900 USD due to the efficiency of these teams [6]. The University of British Columbia implemented a strategy to ensure a consistent team for pediatric spine cases and found a reduction in infections, operating time, length of stay, and blood transfusion volumes post-implementation [7]. These findings highlight the invaluable contributions of nurses and support staff in enhancing the overall quality of care.

While several studies have demonstrated the positive effects of teamwork and dedicated surgical spine teams, there are also studies that have reported minimal or no significant differences in outcomes [2,8,9]. For instance, in our study, anesthesiologists dedicated to a higher number of spine cases did not significantly impact outcomes for ACDF and LF patients. This study validates the findings of Wilson et al., indicating that the volume and experience of the anesthesia provider did not have a significant influence on the likelihood of adverse outcomes for ACDF and LF patients [9]. In the Martin et al. study, though they found notable improvements in procedural efficiency, including reduced OR time, blood loss, and transfusion rates, the overall implementation of such teams did not yield clinically significant differences in outcomes [2].

While not the primary focus of this study, it is worth mentioning that numerous studies have highlighted a higher complication rate among patients treated by low-volume spine surgeons in contrast to those managed by highly experienced surgeons, emphasizing the critical importance of surgical expertise and experience [9,10]. It was also observed that standard-volume surgeons may achieve better outcomes with a dual-surgeon approach, particularly for junior surgeons operating with an experienced colleague [11,12]. A team consisting of two attending surgeons markedly decreased anesthesia duration, surgical time, and blood loss in single-level ACDF procedures, all without an uptick in complications rates [13]. Current literature has shown resident involvement tends to have no significant impact on any complication rates when compared to cases with attending surgeons alone [14]. The involvement of a spine fellow, however, was associated with prolonged procedure duration, yet it did not impact long-term postoperative outcomes; additionally, longer fellow training experience correlated with reduced procedural time, indicating a learning effect [15].

This study is subject to several limitations, like its retrospective design and the potential existence of unmeasured confounding variables. In addition, the study was limited to a single institution and focused on a specific geographic area. This may constrain the extent to which the findings can be applied to a wider population of individuals undergoing spinal surgery. The study period spans from July 1, 2019, to June 30, 2023, a relatively short time frame that may not capture long-term trends or account for potential changes in surgical practices or technologies over a longer period. While the study focuses on specific PACU measures and postoperative outcomes, there may be other clinically relevant outcomes (e.g., patient-reported outcomes, long-term follow-up) that were not included.

## Conclusions

In conclusion, this study provides valuable insights into the minimal impact of anesthesiologist volume on post-operative outcomes. While the study did not find clinically significant differences in outcomes based on anesthesiologists' volumes, recent literature emphasizes the crucial role of teamwork and specialized surgical teams in enhancing efficiency and patient care. Further studies are warranted to explore other variables in anesthesia, nursing, and surgical team workflow that may impact patient postoperative outcomes in spinal surgeries.
